# Characterization of the Avian Mitochondrial-Derived Peptide MOTS-c and Its Potential Role as a Metabolic Regulator

**DOI:** 10.3390/ani15152230

**Published:** 2025-07-29

**Authors:** Xin Shu, Jiying Liu, Bingjie Xu, Hui Wang, Li Liu, Xiaotong Zheng, Jianfei Chen

**Affiliations:** 1 Jiangsu Key Laboratory of Sericultural and Animal Biotechnology, School of Biotechnology, Jiangsu University of Science and Technology, Zhenjiang 212100, China; shu18574525727@163.com (X.S.); liujiying@just.edu.cn (J.L.); xbj2264649431@163.com (B.X.); wanghui163899@163.com (H.W.); liuli_dyx@163.com (L.L.); xiaotzheng@just.edu.cn (X.Z.); 2 Key Laboratory of Silkworm and Mulberry Genetic Improvement, Ministry of Agriculture and Rural Affairs, Sericultural Scientific Research Center, Chinese Academy of Agricultural Sciences, Zhenjiang 212100, China

**Keywords:** poultry, mitochondrial-derived peptide, MOTS-c, RNA-seq, AKT signaling pathway

## Abstract

MOTS-c is a mitochondrial peptide that plays a crucial role in regulating energy metabolism, gene expression, and immune processes. However, current research primarily focuses on mammals like humans and mice, with no reports on avian MOTS-c. This study aimed to identify and characterize MOTS-c coding sequences across major poultry species through bioinformatics analysis and experimental validation. The alignment results showed high sequence similarity in the *MOTS-c* coding regions between avian and mammalian species. However, a single nucleotide deletion was identified in avian sequences at the position corresponding to the fourth amino acid residue of mammalian homologs, resulting in divergent downstream amino acid sequences. Despite this deletion, several residues were conserved across species. Phylogenetic analysis of mRNA sequences grouped pigeons with mammals, while protein sequence analysis revealed that poultry and mammals form separate branches, highlighting the divergence between avian and mammalian MOTS-c sequences. Tissue expression profiling demonstrated widespread distribution of chicken *MOTS-c* across multiple tissues, with the highest expression levels in the heart. Fasting significantly reduced heart *MOTS-c* expression, suggesting potential metabolic regulatory functions. Functional analysis of MOTS-c in primary hepatocytes revealed significant enrichment of the ribosome, oxidative phosphorylation, and key signaling pathways (PI3K-AKT and JAK-STAT) following 24 hours of treatment. Western blot validation confirmed MOTS-c-mediated activation of the AKT signaling pathway. This study represents the first comprehensive characterization of avian MOTS-c, providing critical insights into its evolutionary conservation and its potential functional roles in gene expression and cellular metabolism. Our findings establish a foundation for further investigation into the functions of mitochondrial-encoded peptides in avian species.

## Simple Summary

Mitochondria, key energy-producing organelles, critically depend on mitochondrial-encoded genes. This study systematically compared avian mitochondrial open reading frame of the 12S rRNA-c (MOTS-c) coding/protein sequences and performed phylogenetic analysis. Avian MOTS-c sequences exhibit high similarity but contain a key nucleotide deletion compared with mammals, resulting in divergent downstream amino acids. Chicken *MOTS-c* is ubiquitously expressed, with the highest levels in the heart, and is significantly reduced by fasting. Integrating RNA-seq and molecular assays revealed that the chicken MOTS-c peptide modulates the AKT signaling pathway. These findings establish a foundation for exploring mitochondrial-derived peptides in birds and for informing mitochondrial genetic poultry breeding.

## 1. Introduction

Mitochondria serve as the cell’s powerhouses and sites for aerobic respiration, and they possess distinct genetic material and gene systems. Beyond energy production, they are involved in stress responses, signal transduction, apoptosis, and aging [1]. The mitochondrial genome is crucial for sustaining cellular functions alongside the nuclear genome [2,3], though most mitochondrial proteins are encoded by nuclear genes. Among the relatively few mitochondrial-encoded genes, several open reading frames on mitochondrial ribosomal RNAs can encode relevant proteins. Examples include humanin [4,5], small humanin-like peptides 1 to 6 (SHLPs 1-6) [6], and mitochondrial open reading frame of the 12S rRNA-c (MOTS-c) [7]. These short peptides play crucial regulatory roles in various life processes, such as disease progression, immune response, and energy metabolism [8]. Notably, MOTS-c represents a novel mitochondrial-derived peptide that has been discovered more recently. Encoded by the mitochondrial 12S rRNA, this peptide is involved in the regulation of cellular energy metabolism, gene expression, and immune processes [7,9,10,11,12].

In 2015, MOTS-c was first reported as a bioactive peptide encoded by mitochondrial 12S rRNA, highlighting its role in promoting cellular metabolic homeostasis [7]. Its expression is responsive to diverse energy states and influenced by signaling pathways such as protein kinase B (AKT) and AMP-activated protein kinase (AMPK). Subsequent research by Kim et al. [9] demonstrated that MOTS-c can translocate to the cell nucleus and regulate nuclear gene expression, establishing it as a key auxiliary gene expression regulator. Beyond its metabolic functions, MOTS-c is also involved in lipid metabolism [13,14]. MOTS-c genetic polymorphisms (e.g., m.1382A > C causing Lys14Gln) have been linked to longevity [15]. They may also be associated with male type 2 diabetes onset [16] and are associated with muscle composition and strength [17]. Exercise across mammalian species and human age groups increases MOTS-c expression, enhancing health outcomes [10]. This enhancement occurs through multiple mechanisms, including improved metabolic activity, insulin sensitivity, physical exercise capacity, and cardiovascular function. These effects position MOTS-c as a powerful promoter of healthy aging [18]. Clinically, MOTS-c shows potential for treating diabetes and age-related diseases [19]. It has demonstrated efficacy in alleviating various diseases and injuries [20,21,22,23,24]. Additionally, MOTS-c may regulate bone metabolism [25], enhance cellular resistance to HBV infections [12], and exhibit antibacterial and immune regulatory functions [11]. In summary, MOTS-c is a recently discovered mitochondrial-derived peptide that plays a critical role in multiple biological processes. Its expression is modulated by the AMPK and AKT signaling pathway, varying energy states, and factors like exercise.

Current research on MOTS-c predominantly focuses on mammals such as humans and mice, with no reports available regarding avian species. For poultry, MOTS-c represents an entirely novel gene with unknown functions. Given that mitochondrial rRNA sequences are highly conserved across different species [26], it is hypothesized that avian MOTS-c may possess similar biological roles. In modern large-scale poultry farming, metabolic disorders and diseases seriously affect production efficiency. Identifying key metabolic regulatory genes will offer new targets for healthy poultry farming. Therefore, the primary objectives of this study are to clone the coding sequences (CDS) of MOTS-c in key avian species through homology-based approaches; then to elucidate sequence conservation via comparative alignment and phylogenetic analysis; subsequently, to identify major expression tissues in chickens as a model organism; further, to examine tissue-specific expression dynamics under varying energy states; and finally, to investigate the regulatory mechanisms of chicken MOTS-c peptide on cellular energy metabolism using hepatocytes, integrated with RNA-seq, Western blot, and complementary methodologies.

## 2. Materials and Methods

### 2.1. Animals and Sample Collection

The animal protocols (approval #GQ20230302 dated 1 March 2023; #G2022SJ13 dated 8 March 2022) were approved by the Laboratory Animal Ethics Committee of Jiangsu University of Science and Technology. The animal care and handling practices followed the Committee’s guidelines.

The chicken samples were obtained from a previous study [27]. Eighteen 15-day-old broilers were divided into three groups: fed (free access to food for 18 h), fasted (18 h of starvation), and refed (18 h of fasting followed by 2 h of refeeding). Tissues, including pectoralis, brain, heart, liver, spleen, lung, kidney, proventriculus, gizzard, duodenum, jejunum, ileum, cecum, colon, and skin, were collected from all groups. The tissues from the fed group were analyzed for MOTS-c tissue expression profiles. The pectoralis, brain, heart, liver, kidney, proventriculus, and jejunum samples from all groups were used to assess MOTS-c expression levels under different energy conditions. Fertile eggs from Yao chickens were purchased from the market and incubated in an automatic-controlled incubator (Ruitai Incubation Equipment Development Center, Dezhou, China). The incubator temperature was set at 37.8 °C with a relative humidity of 60%, following standard hatchery protocols. Primary hepatocytes were isolated and cultured from embryonic day 14 (E14) chicken embryos to investigate the regulatory effects of chicken MOTS-c peptide on hepatic energy metabolism.

### 2.2. Predicting Full-Length Coding Sequences of MOTS-c in Different Species

Mitochondrial sequences of common poultry species and some mammals were retrieved from the NCBI database, including chicken (*Gallus gallus*, AB086102.1), quail (*Coturnix japonica*, PP209356.1), turkey (*Meleagris gallopavo*, EF153719.1), duck (*Anas platyrhynchos*, KJ883269.1), goose (*Anser cygnoides*, KY767671.1), pigeon (*Columba livia*, KP168712.1), green monkey (*Chlorocebus sabaeus*, EF597503.1), pig (*Sus scrofa*, MK251046.1), and mouse (*Mus musculus*, KY018919.1). The 12S RNA sequences of each species were extracted according to the mitochondrial genome annotations. Using DNAMAN v6 software, these sequences were aligned with the previously published [7] human (*Homo sapiens*) *MOTS-c* CDS to identify the start codon (ATG) of *MOTS-c* in each poultry species. Subsequently, codon analysis was conducted until the stop codon TAG/TAA was reached, defining the predicted *MOTS-c* sequence for each species.

### 2.3. Phylogenetic Analysis

To investigate the conservation of *MOTS-c* and its cytoplasmic translation protein sequences across species, a phylogenetic analysis of 11 species (6 poultry and 5 mammals) was performed. The evolutionary history was inferred using the neighbor-joining method [28]. The percentage of replicate trees in which the associated taxa clustered together in the bootstrap test (1000 replicates) is shown next to the branches [29]. To *MOTS-c* mRNA sequences, the evolutionary distances were computed using the Tamura 3-parameter method [30] and are in the units of the number of base substitutions per site. To MOTS-c protein sequences, the evolutionary distances were computed using the JTT matrix-based method [31] and are in the units of the number of amino acid substitutions per site. All evolutionary analyses were conducted in MEGA11 [32].

### 2.4. Reverse Transcription Quantitative Real-Time Polymerase Chain Reaction

Total RNA was extracted from each tissue sample using RNAiso Plus reagent (Takara Biotech Co., Ltd., Beijing, China). The RNA quality was assessed via agarose gel electrophoresis, and the concentration was quantified using a Nanophotometer N60 Touch (IMPLEN, Munich, Germany). For cDNA synthesis, 1 μg of total RNA was reverse-transcribed using the Evo M-MLV RT Mix Kit and gDNA Clean reagent (Accurate Biotechnology Co., Ltd., Changsha, China). The resulting cDNA was diluted to a 1:3 ratio with nuclease-free water and stored at −20 °C for subsequent experiments. The reverse transcription quantitative real-time polymerase chain reaction (RT-qPCR) was performed using a Bio-Rad Light Cycler 96 Real-Time PCR system, and a SYBR Green Premix Pro TaqHS qPCR Tracking Kit II (Accurate Biotechnology) was used for the RT-qPCR analysis. The specific reagent components and PCR procedures of RT-qPCR are described in previous studies [27,33]. Based on the cloned chicken *MOTS-c* sequence, RT-qPCR primers were designed using Primer Premier 5.0. Detailed primer sequences are presented in Table S1. The selection of *TBP* and *RPL13* reference genes for tissue gene expression profile analysis was based on previous research [27,34]. To ensure accurate quantification of gene expression across different tissues and between assay plates, a pooled cDNA sample comprising all tissue types was used as a universal reference control for calculating relative expression levels in the RT-qPCR experiments. For RT-qPCR on the chicken primary hepatocytes, *WAC* was chosen as the reference gene [35].

### 2.5. Chicken MOTS-c Synthesis

The chicken MOTS-c peptide was synthesized by Sangon Biotech (Shanghai, China) according to the polypeptide sequence predicted in this study. Supplementary Figures S1 and S2 depict that high-performance liquid chromatography and mass spectrometry were used to confirm the quality and purity of the chicken MOTS-c (95.12%).

### 2.6. Chicken Embryo-Derived Primary Hepatocytes Isolation and MOTS-c Treatment

The chicken primary hepatocytes were isolated using an improved method based on previous descriptions [36,37]. In brief, liver tissue from E14 chick embryos was excised, rinsed two to three times with PBS, and diced into 1–2 mm^3^ fragments using sterile scissors. The tissue was digested with 1% trypsin at 37 °C for 15 min, followed by filtration through cell strainers with pore sizes of 100 μm, 70 μm, and 40 μm to collect the primary hepatocytes. The hepatocytes were cultured in Dulbecco’s Modified Eagle Medium (DMEM) (Gibco, Shanghai, China) supplemented with 1% penicillin/streptomycin at 37 °C with 5% CO_2_. For the MOTS-c treatment experiments, the cells were seeded into 12-well plates (NEST Biotechnology, Wuxi, China). After 12 h of incubation, the medium was replaced and the cells were rinsed with PBS, and then treated with 10 μM and 50 μM MOTS-c in complete DMEM. The concentration of MOTS-c peptide for cell treatment was determined based on a previous study [7].

### 2.7. RNA-Seq and Read Mapping

The primary hepatocytes were treated with 10 μM MOTS-c for 24 h. Total RNA was extracted using the RNAiso Plus reagent (Takara). RNA integrity was assessed using the RNA Nano 6000 Assay Kit on a Bioanalyzer 2100 system (Agilent Technologies, Santa Clara, CA, USA). RNA-seq library preparation, sequencing, and read mapping were performed as previously described [38], using the latest chicken genome (GRCg7b) as the reference. The mapped reads were assembled with StringTie v1.3.1 using a reference-based approach. Transcript expression levels were quantified using RSEM (http://deweylab.github.io/RSEM/ (accessed on 26 January 2025)) by calculating TPM values. Differential expression analysis was conducted using the DESeq2 R package (version 1.30.0).

### 2.8. Gene Set Enrichment Analysis

Gene Set Enrichment Analysis (GSEA) was conducted utilizing the GSEA software (version 4.0.3) and MSigDB database (http://www.gsea-msigdb.org/gsea/msigdb/ (accessed on 26 January 2025)) to determine whether significant differences existed in the gene sets associated with specific GO terms and KEGG pathways between the negative control (NC) and MOTS-c treatment groups, following the methodology outlined by Subramanian et al. [39]. Briefly, the gene expression matrix was input into the software, with genes ranked via the Signal-to-Noise normalization approach. The enrichment scores and *p* values were computed under the default parameters.

### 2.9. Western Blot

The primary hepatocytes were pretreated with 10 or 50 μM chicken MOTS-c (N = 2 per condition) for 8 or 24 h in two independent experiments, yielding four samples total per pretreatment. The cells were lysed using a RIPA buffer containing protease inhibitors. The supernatant was collected after centrifugation at 12,000 rpm for 5 min. The protein concentration in the supernatant was measured using the BCA protein assay kit (Beyotime Biotechnology, Shanghai, China). The proteins were then denatured at 105 °C for 20 min. The protein samples were separated by SDS-PAGE and transferred to a PVDF membrane (Millipore, Shanghai, China). The membrane was blocked for 15 min with Rapid Closure Solution (Beyotime Biotechnology) and incubated with primary antibody overnight at 4 °C. After washing with TBST, the membrane was incubated with secondary antibody for 1 h at room temperature. The following antibodies were used: Anti-β Actin (dilution 1:4000, Catalogue No. ZB15001-HRP-100, Servicebio, Wuhan, China), AKT (dilution 1:1000, Catalogue No. WL0003b, Wanleibio, Shenyang, China), phosphorylated AKT (dilution 1:1000, Catalogue No. WLP001a, Wanleibio), AMPK (dilution 1:10,000, Catalogue No. 10929-2-AP, Proteintech, Wuhan, China), phosphorylated AMPK (1:4000, Catalogue No. WL05103, Wanleibio), and HRP-conjugated Goat Anti-Rabbit (dilution 1:4000, Catalogue No. SA00001-2, Proteintech). Chemiluminescence was detected using an ECL reagent (Abbkine Scientific Co., Ltd., Wuhan, China), and blot bands were visualized with ChemiScope6100 (Clinx Science Instruments Co., Ltd., Shanghai, China). After detecting AKT/AMPK, the membrane was stripped with antibody stripping buffer (Beyotime Biotechnology) and re-probed with phospho-specific antibodies against p-AKT and p-AMPK.

### 2.10. Cell Counting Kit-8 Assay

Cell proliferation was assessed using the Cell Counting Kit-8 (CCK8) (APExBIO, Shanghai, China). The chicken embryo-derived primary hepatocytes were cultured in 96-well plates at 37 °C with 5% CO_2_ and were treated with 10 μM and 50 μM chicken MOTS-c in eight technical replicates per group. After approximately 8 h and 24 h, 10 μL of CCK8 reagent was added to each well, followed by an additional incubation at 37 °C for 2.5 h. Absorbance at 450 nm was measured using a BioTek Epoch 2 microplate auto-reader (BioTek, Winooski, VT, USA).

### 2.11. Statistical Analysis

The raw RT-qPCR data were collected and analyzed using the Bio-Rad CFX96 Manage software (version 4.1). The CT values were exported to Microsoft Excel for further analysis. The relative gene expression levels were normalized using the 2^−△△CT^ method. The gene expression levels were standardized against two reference genes, using the geometric mean of their expression levels to calculate the final expression values, in accordance with published protocols for chicken tissue gene expression analysis [27]. A one-way ANOVA was used to evaluate the statistical significance between different groups, with significance considered at *p* < 0.05.

## 3. Results

### 3.1. Comparison of Gene and Protein Sequences of MOTS-c Across Various Species

**Figure 1:**
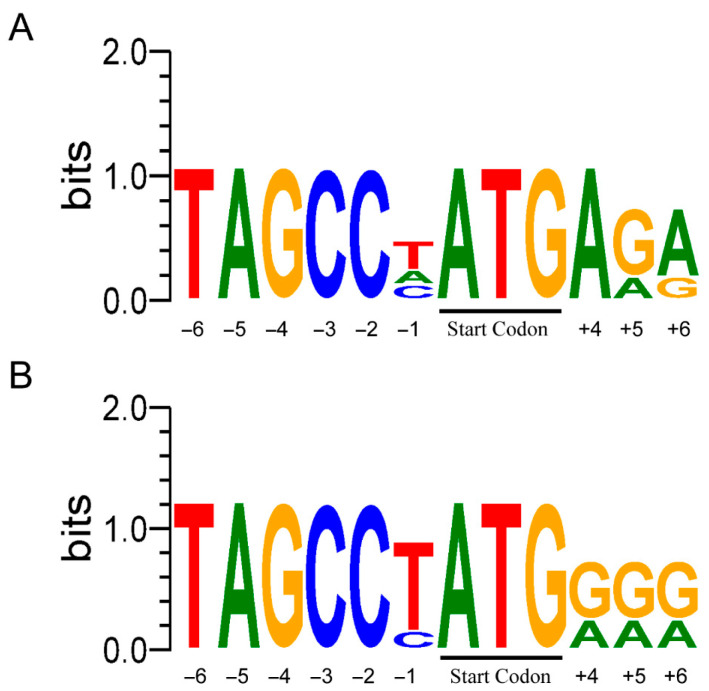
Kozak consensus sequence surrounding the start methionine of *MOTS-c* in mammalian (**A**) and poultry species (**B**). *MOTS-c* sequence logos were generated with WebLogo 3 (https://weblogo.threeplusone.com/ (accessed on 3 November 2024)) [40].

**Figure 2:**
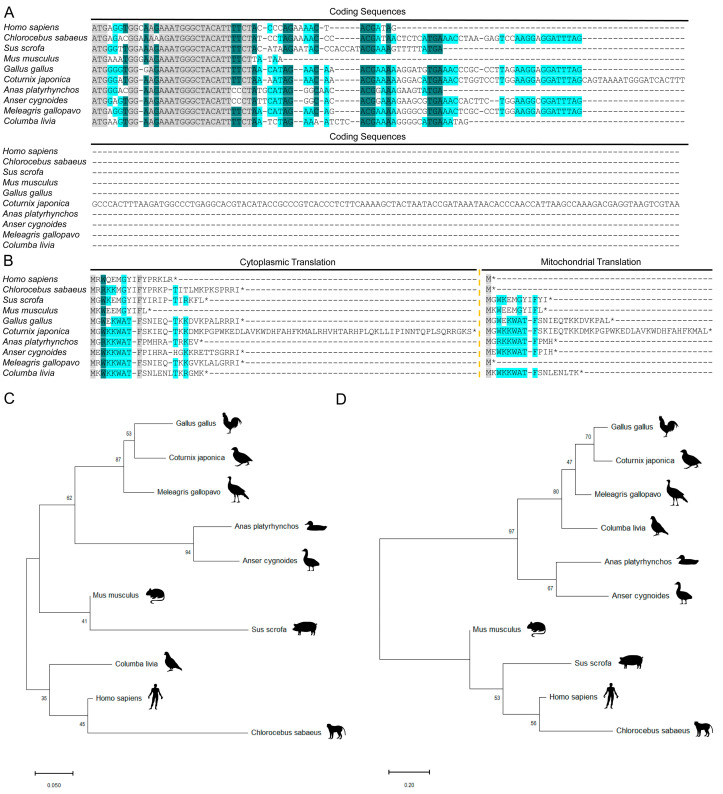
Multiple sequence alignments and phylogenetic analysis of MOTS-c sequences in different species. (**A**,**B**) represent multiple sequence alignments of MOTS-c mRNA and protein sequences in different species. (**C**,**D**) represent the phylogenetic tree of the mRNA and protein sequences of MOTS-c (including cytoplasmic and mitochondrial translation) in different species, respectively. Gray color indicates identical amino acids across species, deep blue over 80% similarity, and green at least 50% similarity. An asterisk (*) marks the translation stop site in MOTS-c across species. Silhouettes of vertebrates were available under public domain licenses at PhyloPic (http://phylopic.org/ (accessed on 17 December 2024)).

The consensus sequence depicted in Figure 1 indicates that the predicted *MOTS-c* in poultry exhibits greater conservation with the Kozak sequence, particularly at a position one nucleotide downstream from the start codon, where most avian species have a G. This suggests that the predicted poultry *MOTS-c* also holds potential for protein translation. The alignment of the cytoplasmic coding sequences of predicted *MOTS-c* across various species reveals a high degree of conservation among different organisms (Figure 2A). However, sequence alignment reveals that avian *MOTS-c* has a single base deletion at the fourth codon compared to mammals, leading to obvious divergence in the amino acid sequence of avian MOTS-c starting from the fourth residue relative to mammalian counterparts (Figure 2A,B). Despite this, some core sequences of the cytoplasmic proteins remain conserved. Among the predicted cytoplasmic MOTS-c proteins, that of the quail is the longest (Figure 2B). Additionally, given the difference in mitochondrial stop codons compared to those in the cytoplasm, we also predicted mitochondrial MOTS-c for different species, which are generally shorter: human, green monkey, and turkey mitochondrial MOTS-c encode just one amino acid (Figure 2B). Phylogenetic analysis of mRNA sequences shows that avian *MOTS-c* predominantly cluster together, yet the pigeon sequence appears within the mammalian clade (Figure 2C), indicating relatively minor differences between mammalian and avian *MOTS-c* mRNA sequences. Analysis of the phylogenetic tree based on predicted cytoplasmic MOTS-c proteins reveals that all avian MOTS-c cluster together and form distinct branches separate from mammalian lineages (Figure 2D).

### 3.2. Chicken MOTS-c Gene Expression Profile and Its Association with Different Energy States

**Figure 3:**
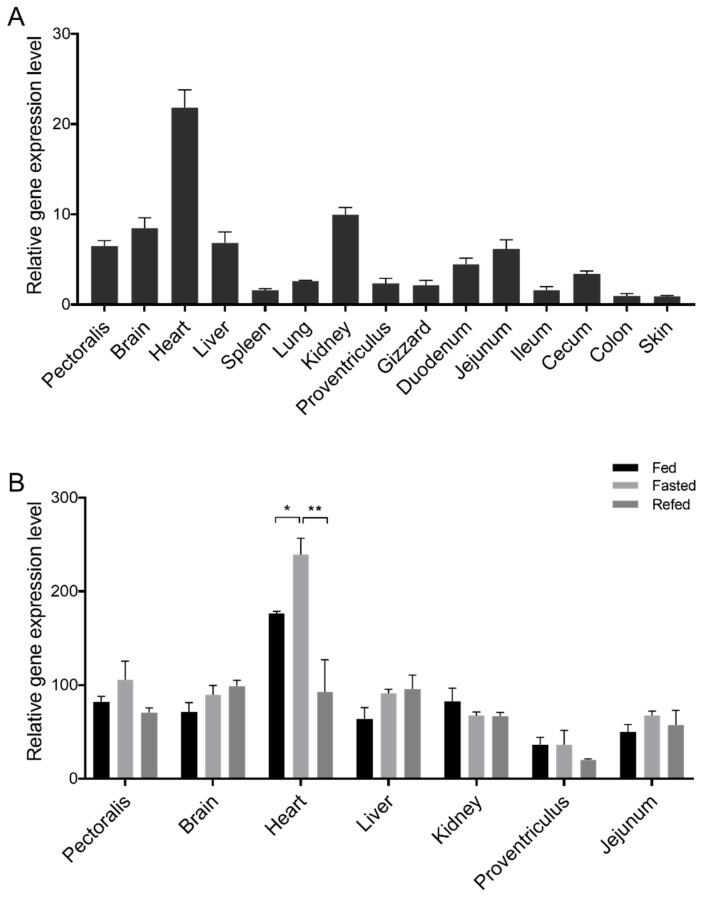
Relative expression levels of chicken *MOTS-c*. (**A**) Relative gene expression profiles of chicken *MOTS-c* in 15 tested tissues. (**B**) Gene expression levels of chicken *MOTS-c* with different energy states. In the RT-qPCR assays, *TBP* and *RPL13* were employed as reference genes. A pooled cDNA sample comprising all tissue types served as the universal reference control for calculating *MOTS-c* expression levels. Each experimental group consisted of six biological replicates, with three technical replicates performed per sample. * indicates significant differences (*p* < 0.05). ** indicates highly significant differences (*p* < 0.01).

To investigate the gene expression characteristics of *MOTS-c* in poultry, we selected chickens as a model organism and examined *MOTS-c* expression profiles across major tissues. Chicken *MOTS-c* is expressed across multiple tissues, with notably higher expression levels in the muscle, brain, heart, liver, kidney, and jejunum, and the highest expression observed in the heart (Figure 3A). Furthermore, we evaluated how different energy levels affect *MOTS-c* expression in tissues with high baseline expression. The findings demonstrated that chicken *MOTS-c* exhibited a significant response to energy states (Figure 3B). Under fasting conditions, the expression level of *MOTS-c* in the heart exhibited a significant increase in comparison to the fed group and the refed group. The same trend was observed in muscle tissue, but the difference was not significant (*p* = 0.24). In liver tissue, the expression levels of *MOTS-c* in the fasting group and the refed group were higher than in the fed group, although the difference was not statistically significant (*p* = 0.093).

### 3.3. Functional Role of Chicken MOTS-c Peptide in Liver Metabolism: An RNA-Seq Analysis

**Figure 4:**
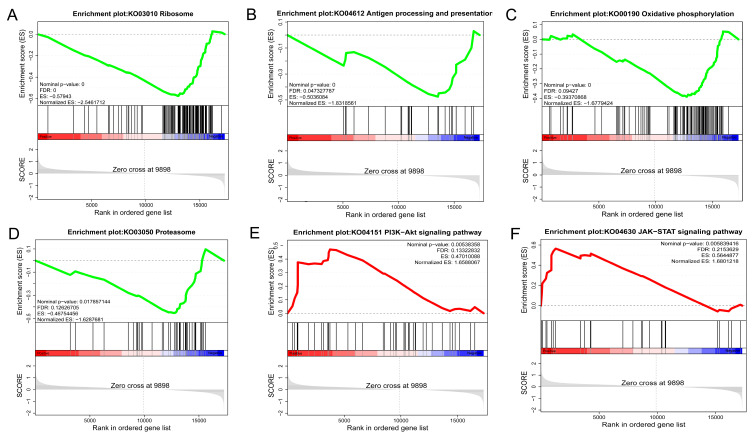
GSEA KEGG enrichment results of RNA-seq data from primary chicken embryo hepatocytes treated with chicken MOTS-c for 24 h. (**A**–**F**) represent the specific GSEA KEGG pathways enriched.

To investigate MOTS-c’s function, primary chicken embryo liver cells were treated with exogenous MOTS-c peptide for 24 h before RNA-Seq experiments. Each library yielded 5.75–6.96 Gb of clean reads (with a mean of 6.53 Gb for the NC group and 6.54 Gb for the MOTS-c treatment group). The data quality was high (Q20 > 97.90%, Q30 > 94.92%), with other quality metrics shown in Table S2. Per library, 90.63–91.52% of the reads uniquely mapped to the chicken reference genome (Table S3). Following assembly, we identified 17,300 genes. By applying |log2FoldChange| > log2(1.5) and *p* < 0.05, we selected sixteen differentially expressed genes (DEGs) (nine upregulated, seven downregulated) (Figure S3; Table S4). To validate RNA-Seq reliability, five randomly selected DEGs underwent RT-qPCR verification. The results demonstrated notable concordance with transcriptomic data (Table S5), supporting the robustness of our sequencing analysis. Despite few DEGs and no significant GO/KEGG enrichments, the GSEA analysis highlighted significant enrichment of pathways including ribosome, oxidative phosphorylation, antigen processing and presentation, proteasome, PI3K-AKT signaling pathway, and JAK-STAT signaling pathway following MOTS-c treatment (Figure 4A–F).

### 3.4. The Effect of Chicken MOTS-c Peptide Treatment on the AKT and AMPK Signaling Pathways

**Figure 5:**
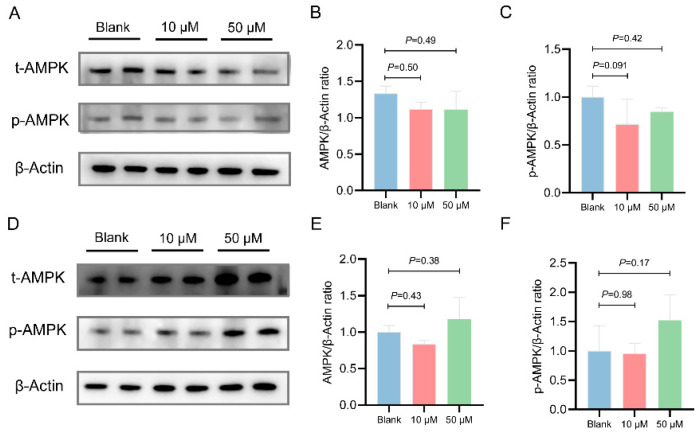
Effects of chicken MOTS-c on AMPK signaling in chicken embryo-derived primary hepatocytes. Primary hepatocytes were pretreated with 10 or 50 μM chicken MOTS-c (N = 4) for 8 or 24 h. (**A**,**D**) Representative protein blots of AMPK, p-AMPK, and actin at 8 h and 24 h, respectively. (**B**,**C**) and (**E**,**F**) Quantification of AMPK and p-AMPK protein levels at 8 h and 24 h, respectively.

**Figure 6:**
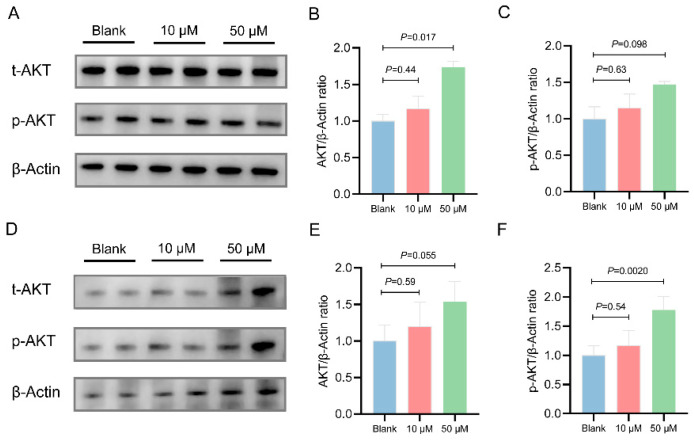
Effects of chicken MOTS-c on AKT signaling in chicken embryo-derived primary hepatocytes. Primary hepatocytes were pretreated with 10 or 50 μM chicken MOTS-c (N = 4) for 8 h or 24 h. (**A**,**D**) Representative protein blots of AKT, p-AKT, and actin at 8 h and 24 h, respectively. (**B**,**C**) and (**E**,**F**) Quantification of AKT and p-AKT protein levels at 8 h and 24 h, respectively.

To further verify the regulatory effects of chicken MOTS-c on the AMPK and AKT signaling pathways, we treated the chicken primary embryonic hepatocytes with MOTS-c peptide for 8 h and 24 h. After 8 h of MOTS-c treatment, no significant differences in AMPK or p-AMPK levels were observed compared to the control group (Figure 5A–C). At the 50 μM concentration, MOTS-c significantly increased AKT expression (*p* = 0.017) but not p-AKT expression (*p* = 0.098) (Figure 6A–C). After 24 h of MOTS-c treatment, 50 μM MOTS-c increased p-AMPK levels, but not significantly (*p* = 0.17) (Figure 5D,F), while significantly increasing p-AKT expression (*p* = 0.0020) and showing a non-significant increase in AKT expression (*p* = 0.055) (Figure 6D–F). Furthermore, the CCK8 assay of primary chicken embryo liver cells treated with MOTS-c showed no significant differences in cell proliferation across different treatment durations (Figure S4).

## 4. Discussion

The significance of mitochondrial-encoded genes during the long-term co-evolution of mitochondrial and nuclear genes has become increasingly evident in biological research. MOTS-c, a mitochondrial-derived peptide discovered in recent years, holds particular importance in cellular metabolism [7]. In this study, the predicted avian *MOTS-c* sequence exhibits high similarity to its mammalian counterpart. Sequence analysis around the start codon indicates that avian MOTS-c also has the potential for protein translation (Figure 1). Although it partially deviates from the Kozak consensus sequence, different species may exhibit distinct nucleotide preferences in translation initiation. Mitochondria and the nucleus use different genetic codes. In humans, mitochondria interpret “AGA” and “AGG” as stop codons, while nuclear DNA codes them as arginine [41]. Due to the mitochondrial genetic code limitations, human *MOTS-c* translation in mitochondria would be truncated [7]. This is also likely true for turkey *MOTS-c*, suggesting that its translation may predominantly occur in the cytoplasm. However, we observed that the predicted mitochondrial MOTS-c peptides in most avian species, pigs, and green monkeys are relatively longer (Figure 2B). The functional roles of these mitochondrially translated peptides will require further investigation in future studies.

The mitochondrial genome evolves at a faster rate compared to the nuclear genome, likely due to its higher mutation rate and replication rate [42,43,44]. The *MOTS-c* multi-sequence alignment results revealed that the CDS region of avian *MOTS-c* contains a single-base deletion at the fourth amino acid position compared to mammals, resulting in a frameshift mutation (Figure 2A). Consequently, the protein sequences of avian MOTS-c and mammalian MOTS-c diverge significantly. However, several amino acid positions remain highly conserved across species (Figure 2B), leading us to hypothesize that these conserved residues may play critical roles in MOTS-c’s biological functions. Due to the substantial differences in amino acid sequences, this study did not employ existing human antibodies for protein-level investigations. Future research on avian MOTS-c should extend to the protein level, requiring specific antibody design and experimental validation.

To investigate the function of avian MOTS-c, we used chickens as a model. Tissue gene expression profiling showed high *MOTS-c* expression in muscle-rich tissues (heart, muscle, small intestine) and the liver (Figure 3A). Notably, heart *MOTS-c* expression, the highest among all tissues, was significantly affected by energy status (Figure 3B), underscoring its role in energy metabolism regulation. Studies indicate that MOTS-c serves as a critical regulator of mitochondrial health, counteracting the cardiac energy imbalance associated with cardiovascular diseases [45,46]. Unlike in mammals, chicken *MOTS-c* mRNA levels rose after fasting compared to ad libitum feeding. In mice, fasting reduced MOTS-c protein levels [7], possibly due to mRNA–protein translation inconsistencies. Although we could not directly detect chicken MOTS-c protein due to a lack of specific antibodies, the rapid response to energy changes indirectly suggests its existence, warranting further research.

Since the liver plays a crucial role in lipid metabolism in chickens [47,48], and *MOTS-c* is highly expressed there, this study used chicken embryo-derived primary hepatocytes to explore MOTS-c’s function. RNA-seq analysis indicated that chicken MOTS-c significantly activates the PI3K-AKT signaling pathway, a finding confirmed by Western blot (Figure 5) and aligning with human studies [7]. Despite significant sequence divergence from mammalian MOTS-c, chicken MOTS-c can still activate the AKT signaling pathway. This implies that MOTS-c’s functional role is conserved across species, possibly due to partially conserved amino acid sequences (Figure 2B). Moreover, RNA-seq analysis revealed the enrichment of not only various metabolic pathways but also numerous immune-related pathways: antigen processing and presentation, proteasome, and JAK-STAT signaling pathway (Figure 4B,D,F). This agrees with prior studies linking MOTS-c peptides to immunity [11,12], further indicating that chicken MOTS-c shares functional similarities with its mammalian counterpart. These results suggest that chicken MOTS-c may have physiological roles, but the functional mechanisms of avian MOTS-c need further study.

Previous studies indicated that exogenous MOTS-c could markedly suppress ovarian cancer cell proliferation [49]. However, in this study, the CCK8 assay showed no significant effect of exogenous chicken MOTS-c on cell proliferation (Figure S4). Yet, a slight inhibitory trend was observed at 24 h with 50 μM MOTS-c treatment. This discrepancy might stem from differences in cell models. Future experiments should explore the effects of avian MOTS-c on cell proliferation and metabolism using other cancer cell lines or muscle cells.

The study of MOTS-c in mammals has revealed its connections to muscle activity, lifespan, and immunity [18,50,51]. Future poultry research will likely focus more on the links between MOTS-c and economically important traits such as meat quality, egg production, and disease resistance. Similar to human research on MOTS-c polymorphisms [17], future studies should further explore the relationship between *MOTS-c* gene polymorphisms and various production traits in poultry. Some studies have reported that MOTS-c exerts its physiological functions through multiple signaling pathways or downstream proteins [23,52,53], and exercise can regulate MOTS-c secretion [54], offering references for in-depth research on MOTS-c in poultry. Another major research direction is to identify the regulatory factors of MOTS-c expression.

In addition to MOTS-c, there are many other mitochondrial-derived peptides, such as humanin and SHLPs 1-6. However, due to interspecies variability in mitochondrial-derived peptides [55], extensive investigations are still required to elucidate avian mitochondrial-derived peptides. The mitochondrial-derived peptides have a wide range of functions in metabolic regulation, cell protection, disease intervention, and anti-aging within mammals [8,56,57]. However, research on mitochondrial-derived peptides in birds has been notably limited. To date, only a single bioinformatics study has indicated the existence of *humanin* and *SHLP6* in birds [58], yet no in-depth functional exploration has been carried out. In this study, we systematically identified the coding sequence of MOTS-c within avian 12S RNA and performed relevant experimental validation. Our research provides a valuable example for the cloning and functional exploration of other mitochondrial-derived peptides in poultry.

## 5. Conclusions

This study successfully generated full-length coding sequences of poultry MOTS-c. Furthermore, through phylogenetic tree analysis and protein homology alignment, we explored the genetic characteristics of MOTS-c across major poultry species, revealing its conservation and uniqueness among different species. Gene expression analysis indicated that chicken *MOTS-c* responds rapidly to varying energy levels. Transcriptomic analysis in hepatocyte models revealed that MOTS-c plays a key role in regulating energy metabolism, immunity, and oxidative stress. Moreover, treating primary hepatocytes with MOTS-c peptide activated the AKT/AMPK signaling pathway. These findings suggest that chicken MOTS-c is a candidate gene involved in energy metabolism regulation. Overall, our study provides a foundation for further research on poultry MOTS-c functions and offers new candidate genes for studying energy metabolism.

## Data Availability

The RNA-seq data have been deposited in the Genome Sequence Archive in the National Genomics Data Center (GSA: CRA025228), which is publicly accessible at https://ngdc.cncb.ac.cn/gsa (accessed on 27 July 2025).

## Supplementary Materials

The following supporting information can be downloaded at: https://www.mdpi.com/article/10.3390/ani15152230/s1, Table S1: Primers used for RT-qPCR analysis; Table S2: Summary of sample RNA-seq data quality; Table S3: The total reads and mapping results of RNA-seq data; Table S4: Detailed information on differentially expressed genes derived from RNA-seq data; Table S5: RT-qPCR validation of RNA-seq identified differentially expressed genes; Figure S1: The HPLC test result of chicken MOTS-c; Figure S2: The MS test result of chicken MOTS-c; Figure S3: Differentially expressed genes identified using RNA-seq analysis in MOTS-c-treated chicken embryo primary hepatocytes; Figure S4: Effect of chicken MOTS-c on cell proliferation in primary embryo hepatocytes; Figure S5: The original Western blot image of Figure 5A; Figure S6: The original Western blot image of Figure 5D; Figure S7: The original Western blot image of Figure 6A; Figure S8: The original Western blot image of Figure 6D.
