# Supplementary material for: Characterization of the Avian Mitochondrial-Derived Peptide MOTS-c and Its Potential Role as a Metabolic Regulator
Source: Animals (Basel). 2025 Jul 29;15(15):2230. doi: 10.3390/ani15152230 (PMC12345487; doi:10.3390/ani15152230)

Figure S5 - marked

The original Western blot image of Figure 5A. The red box indicates the image area presented in the manuscript. Samples on both sides of the marker are biological replicates from separate batches.

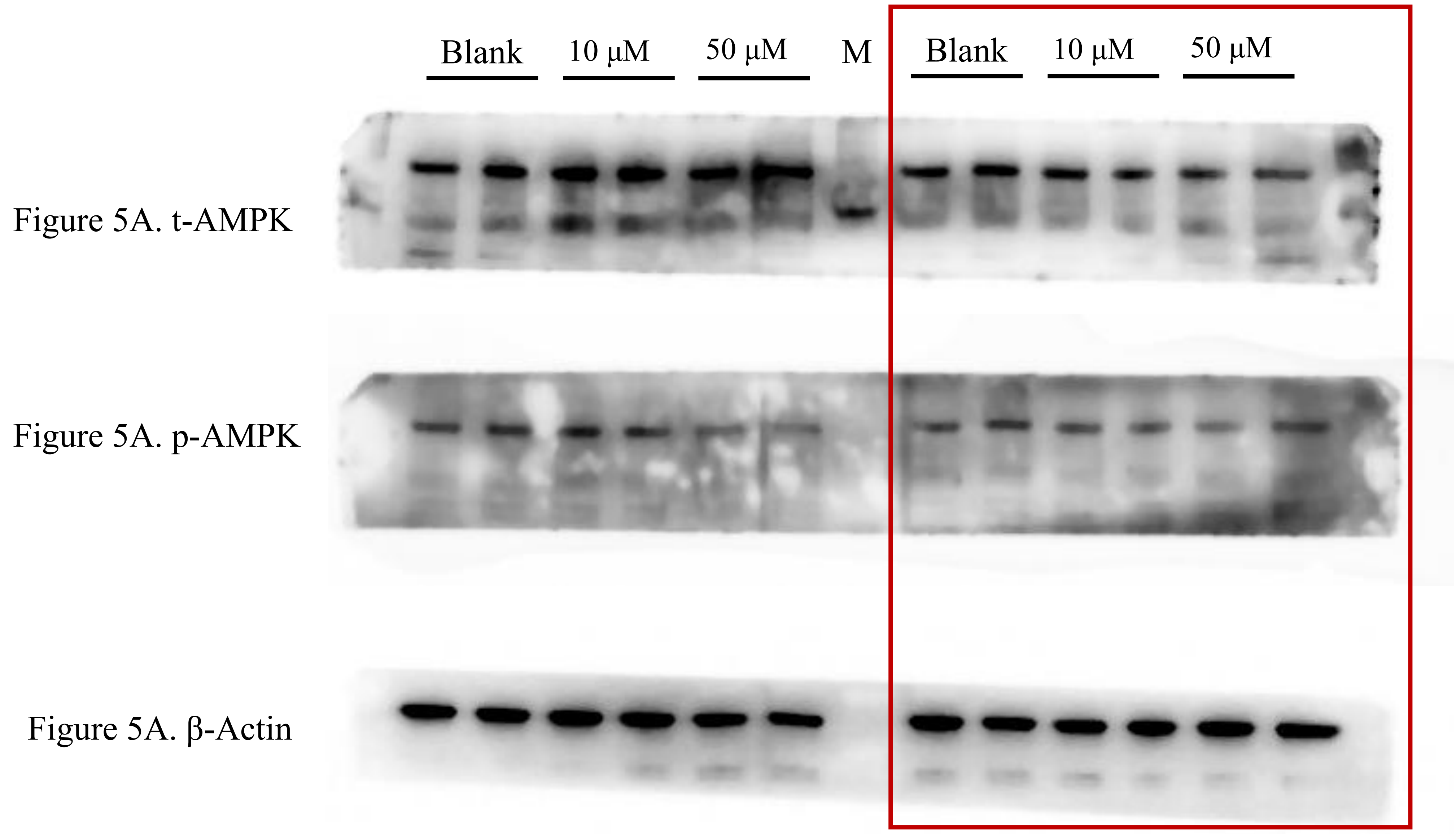

Full membrane Western blot of MOTS-c peptide treatment for 8 hours

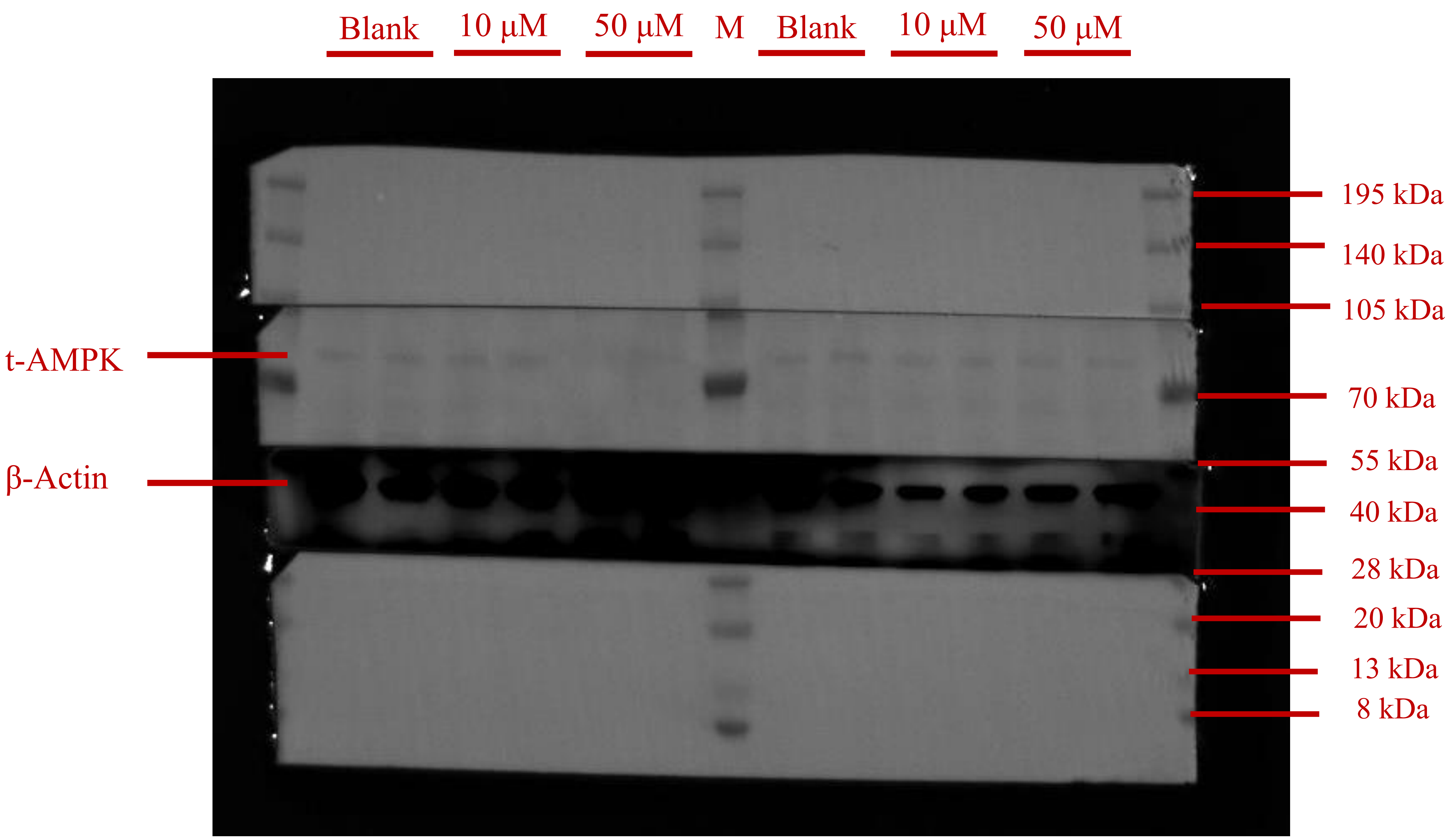

Figure S5 - unmarked

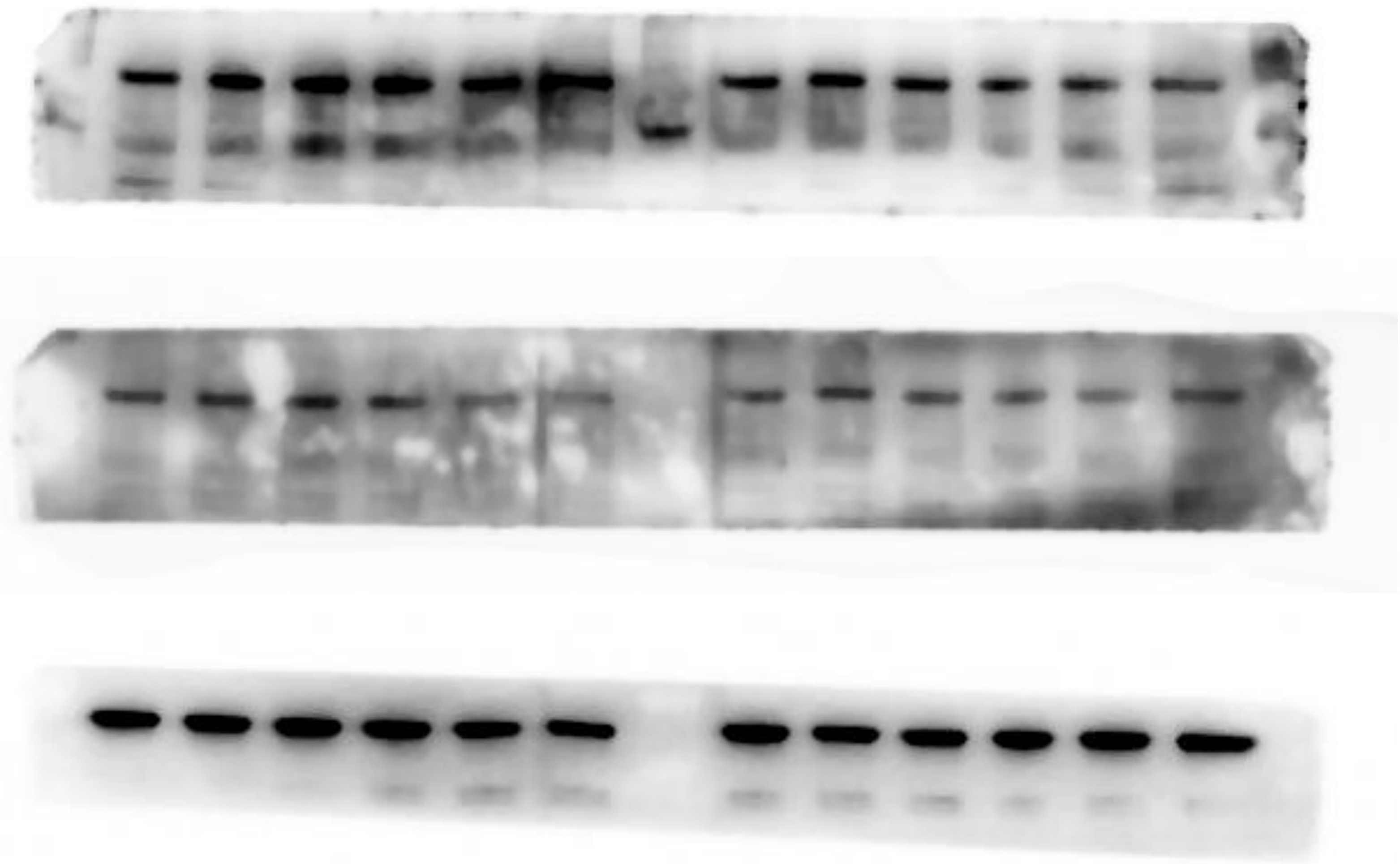

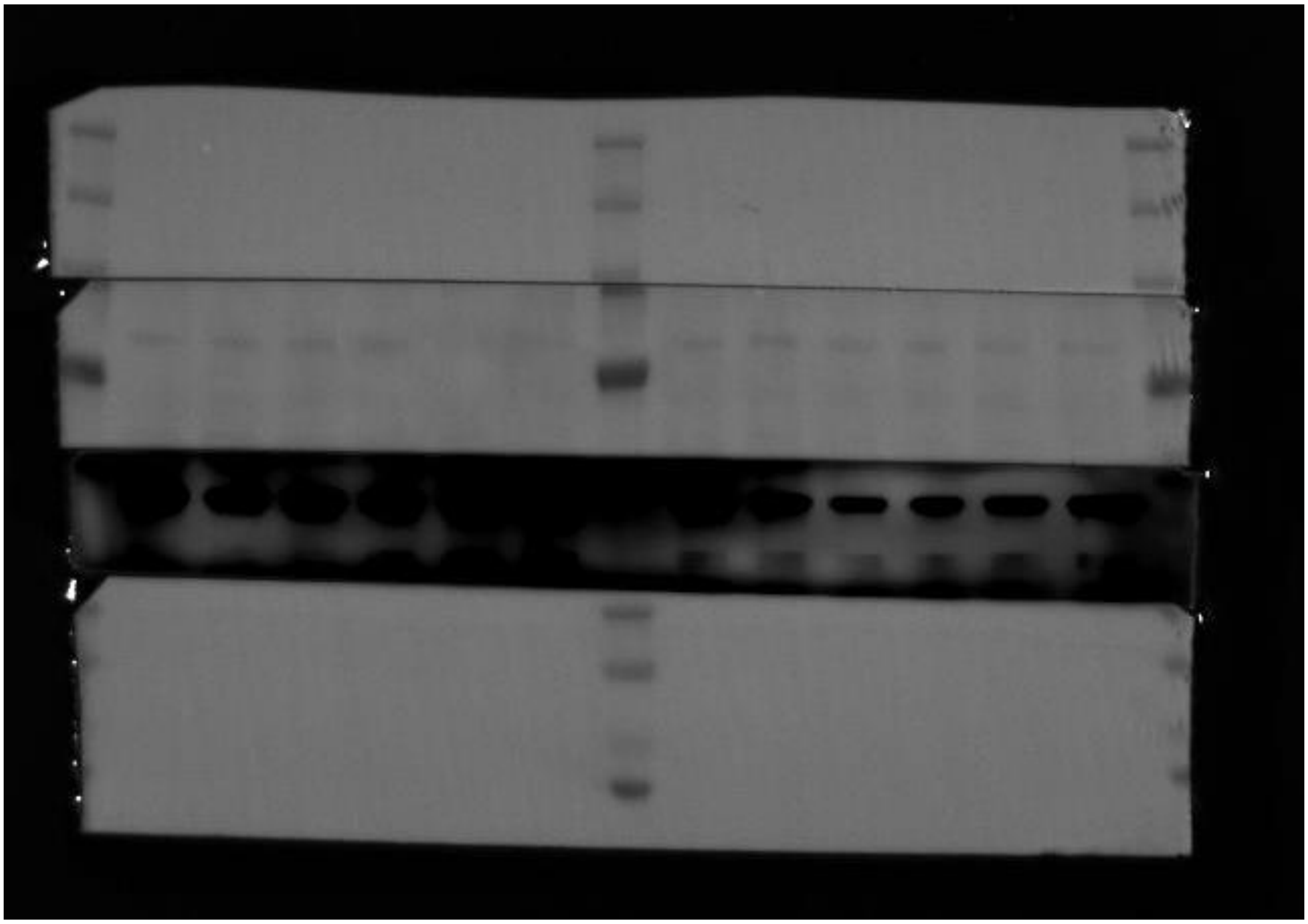

Figure S6 - marked

The original Western blot image of Figure 5D. The red box indicates the image area presented in the manuscript. Samples on both sides of the marker are biological replicates from separate batches.

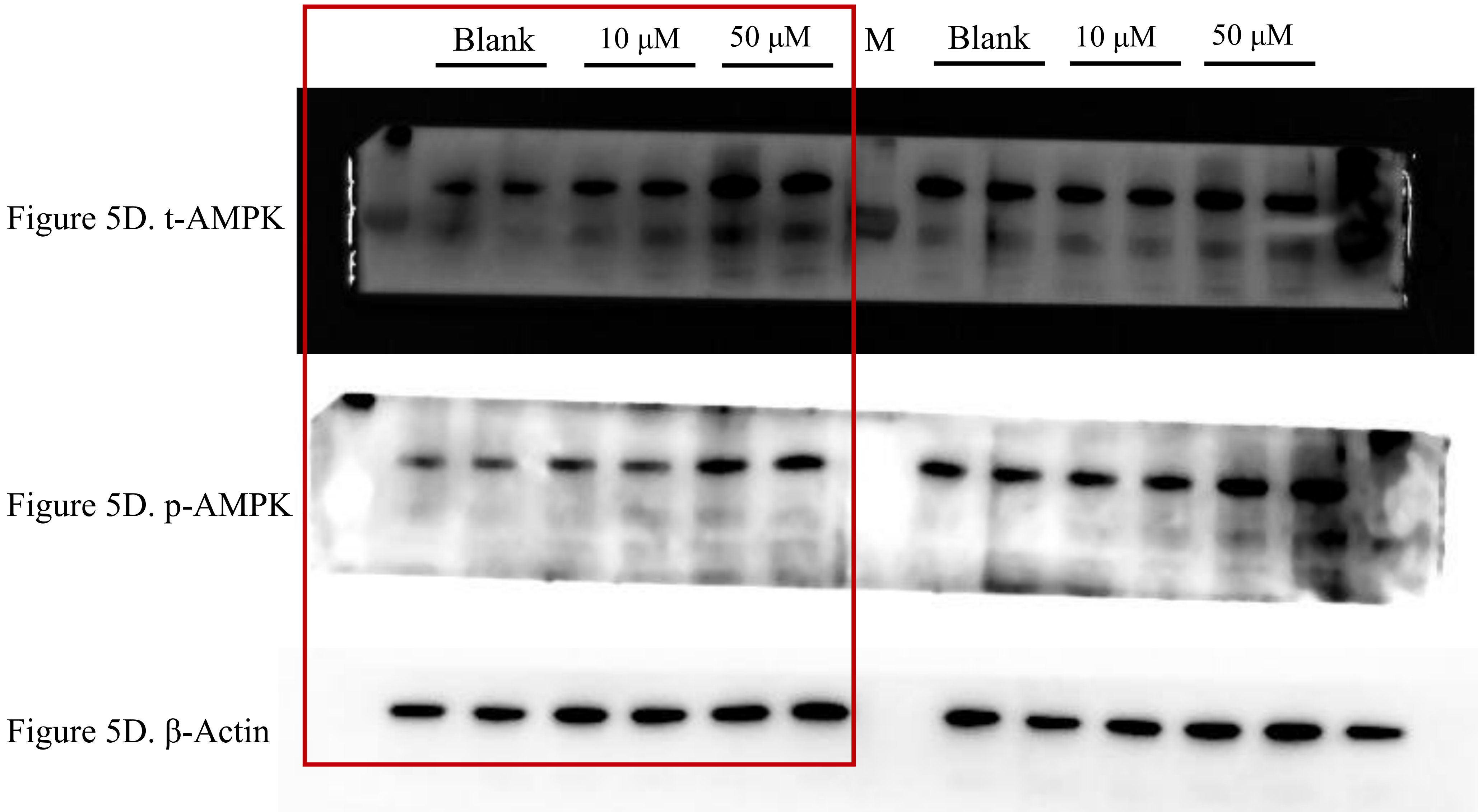

Full membrane Western blot of MOTS-c peptide treatment for 24 hours

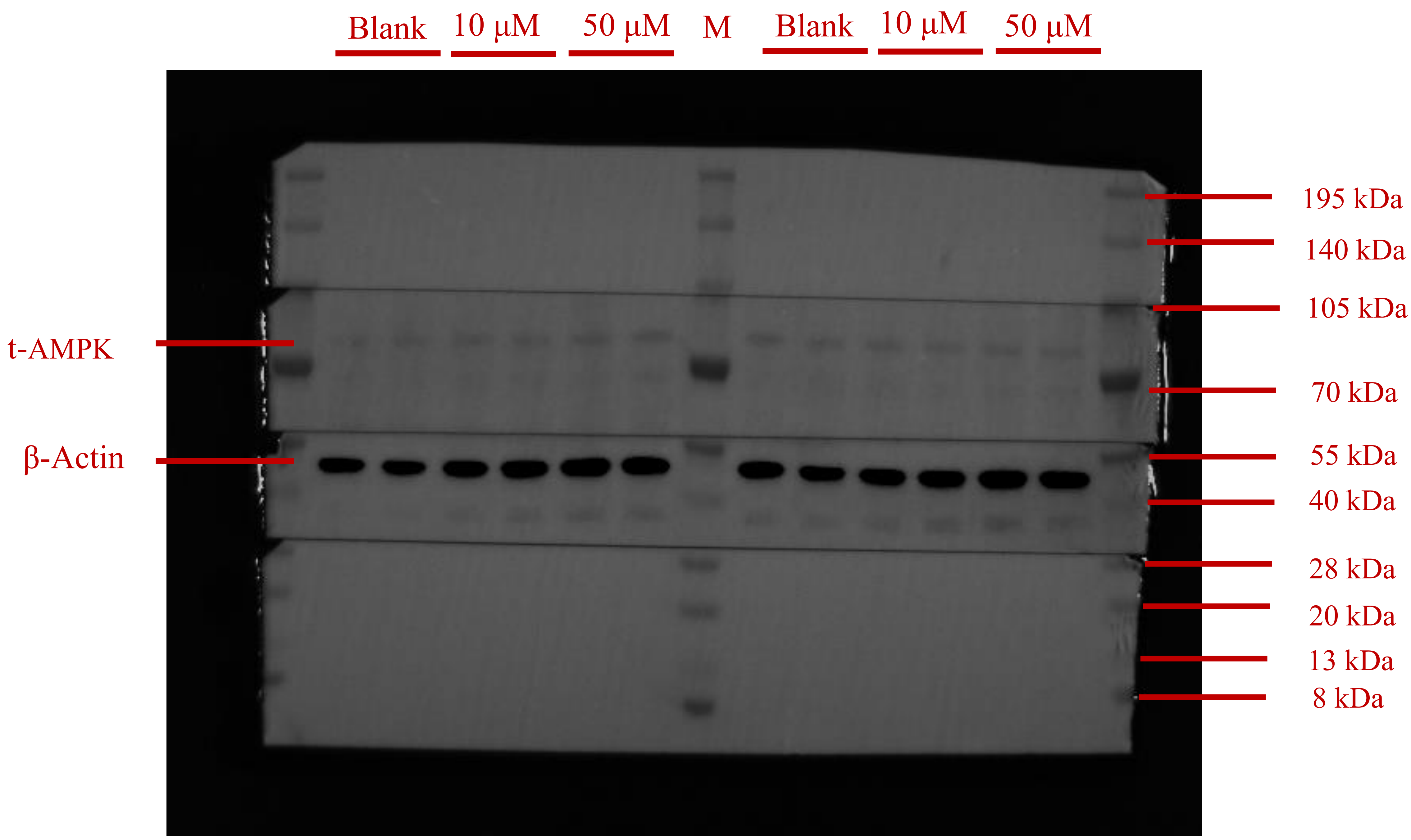

Figure S6 - unmarked

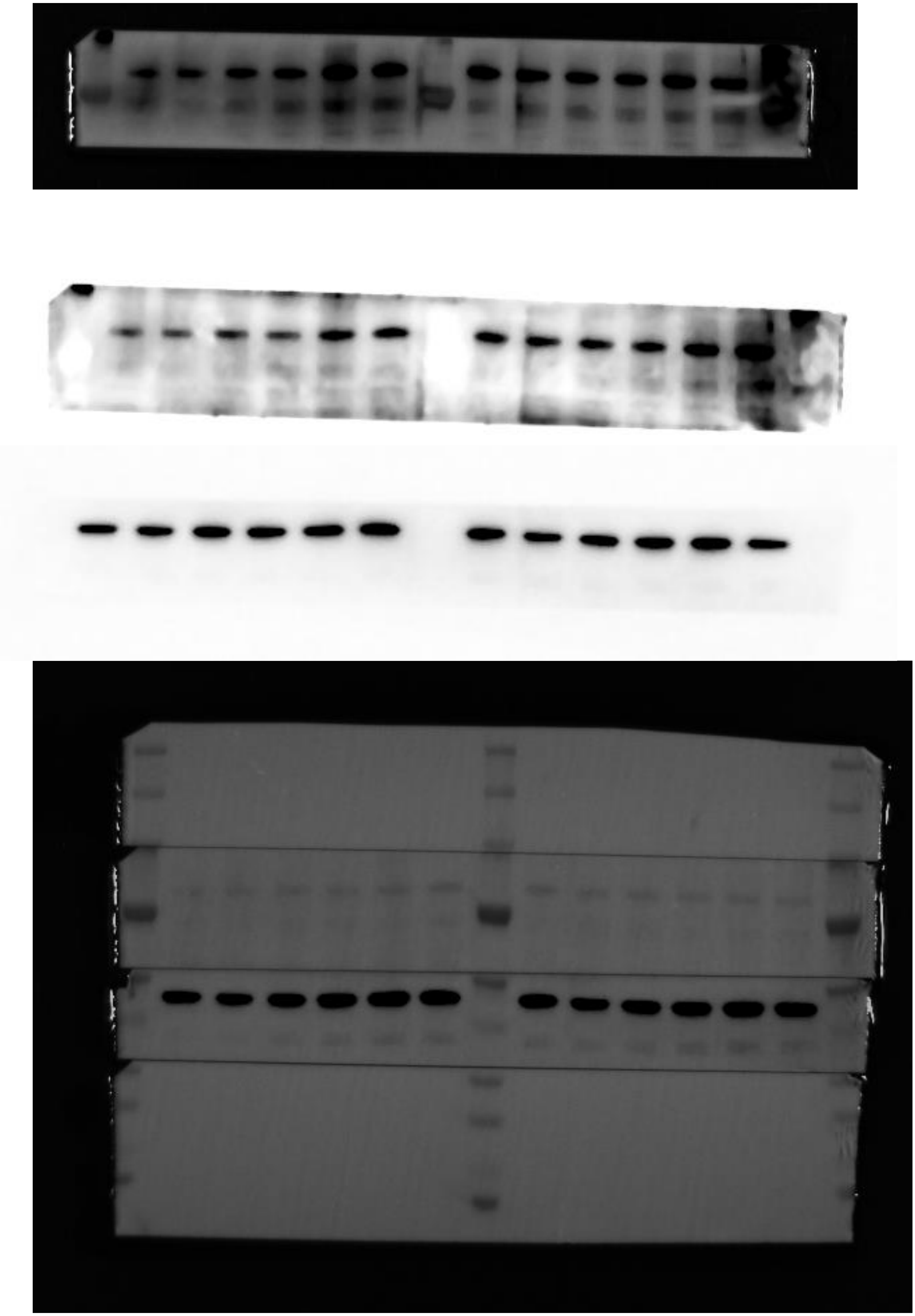

Figure S7 - marked

The original Western blot image of Figure 6A. The red box indicates the image area presented in the manuscript. Samples on both sides of the marker are biological replicates from separate batches.

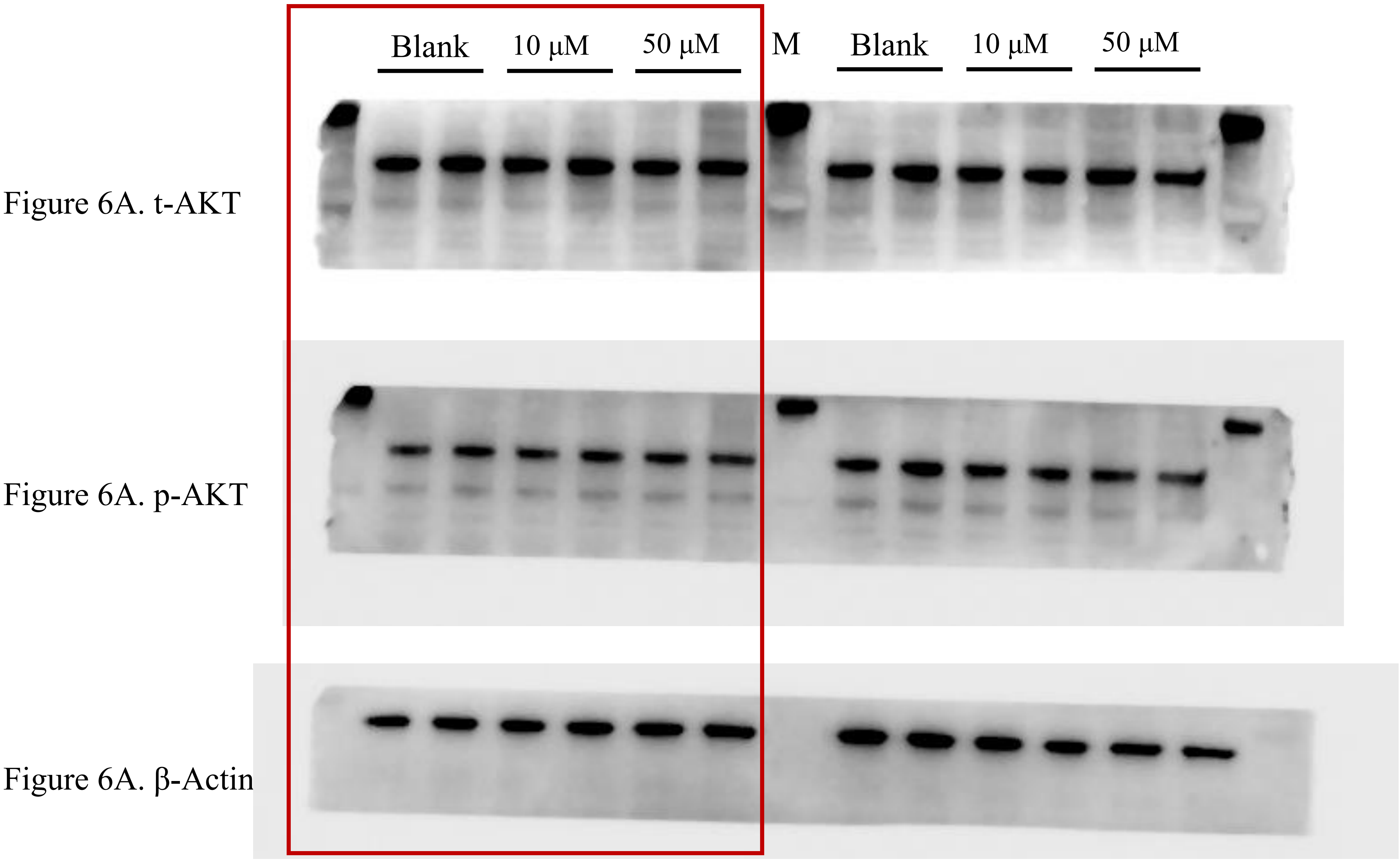

Full membrane Western blot of MOTS-c peptide treatment for 8 hours

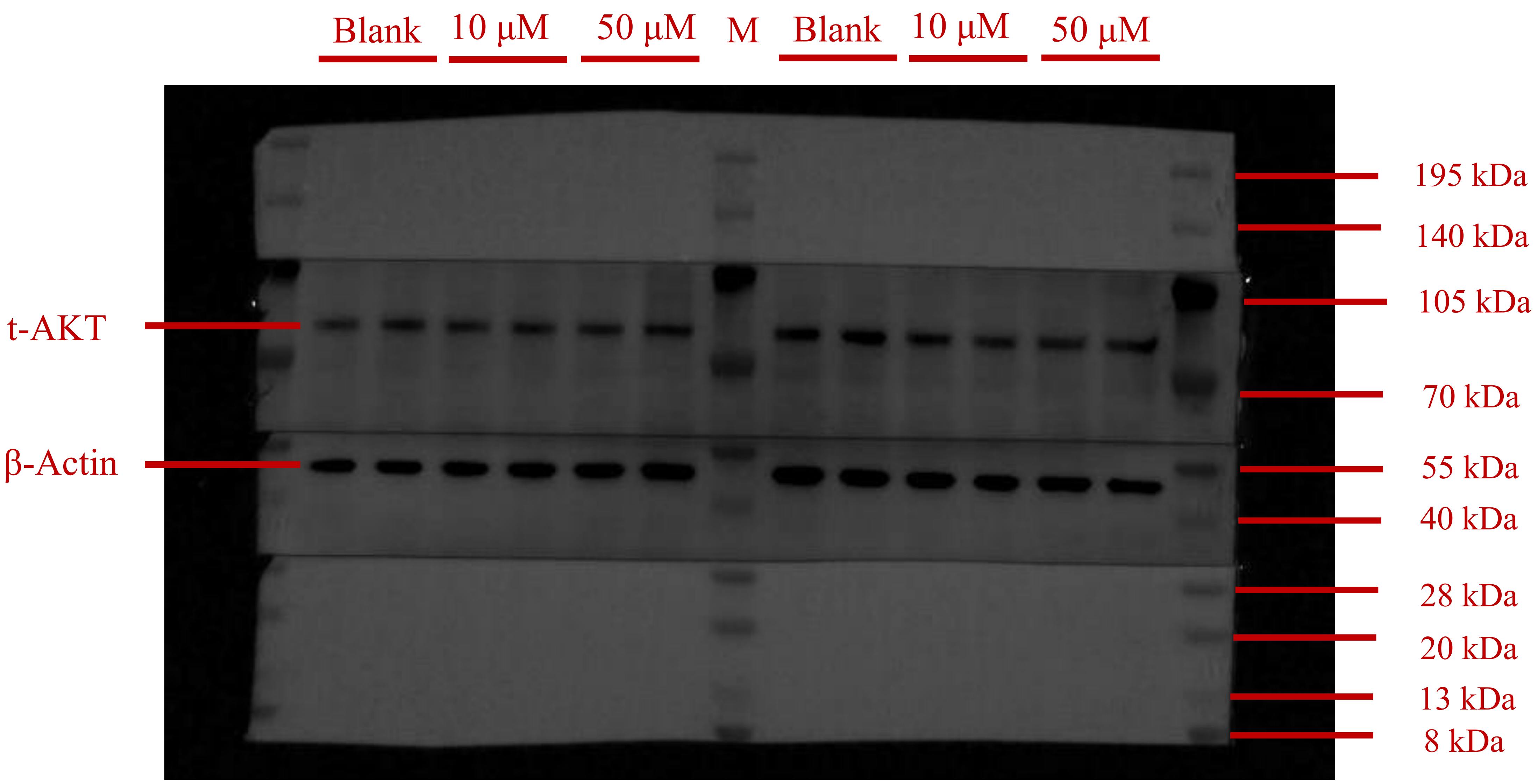

Figure S7 - unmarked

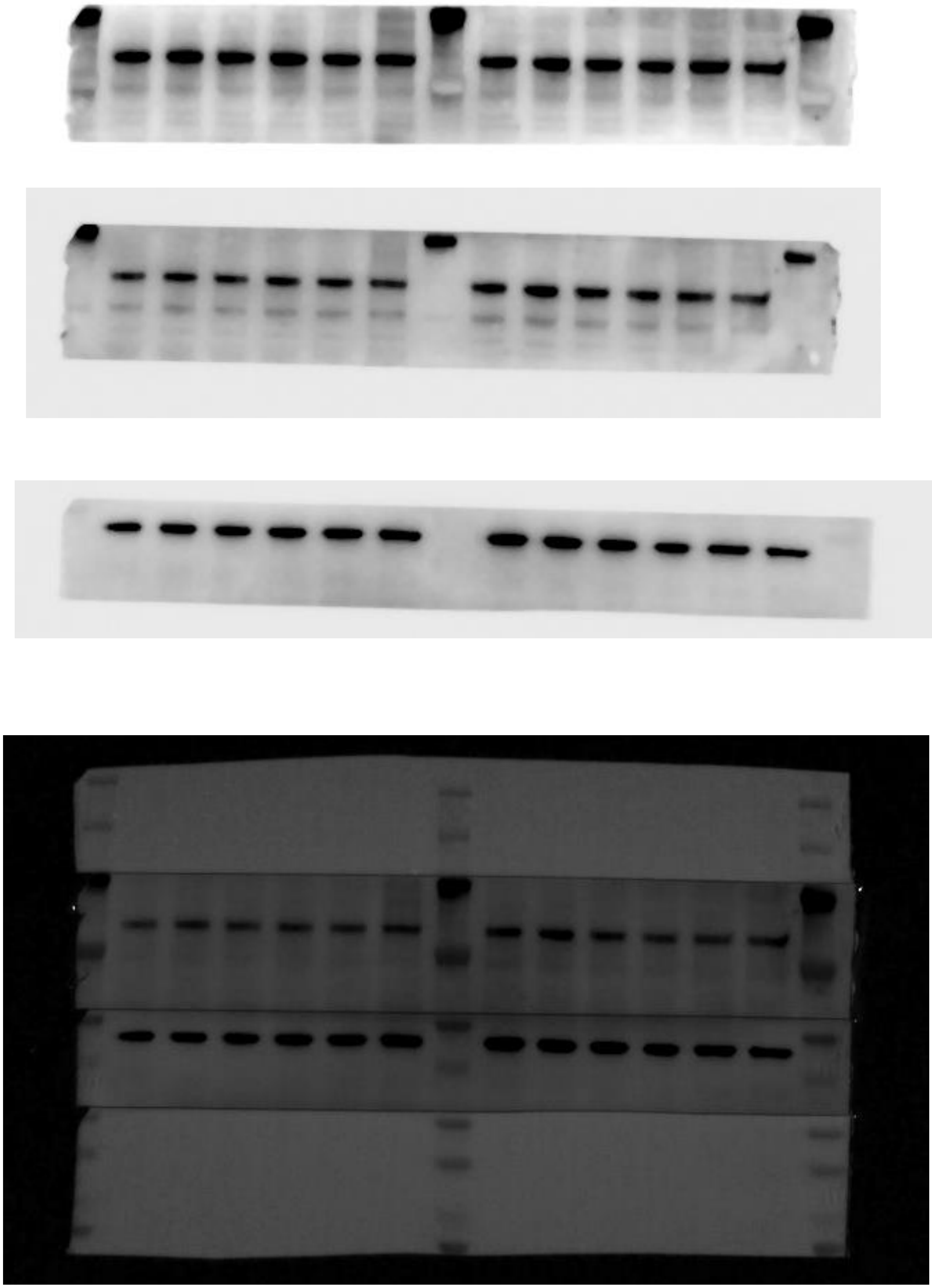

Figure S8 - marked

The original Western blot image of Figure 6D. The red box indicates the image area presented in the manuscript. Samples on both sides of the marker are biological replicates from separate batches.

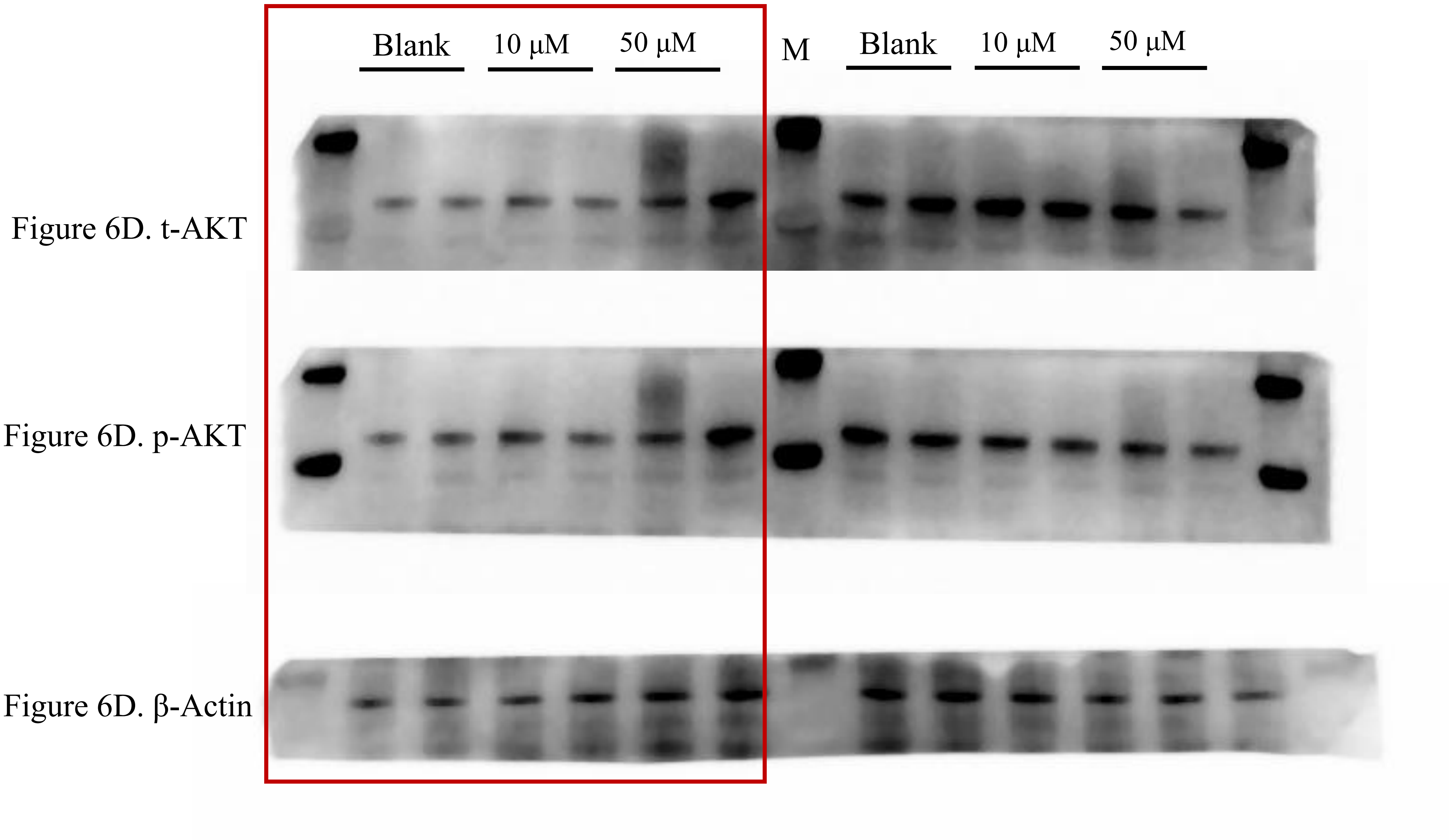

Full membrane Western blot of MOTS-c peptide treatment for 24 hours

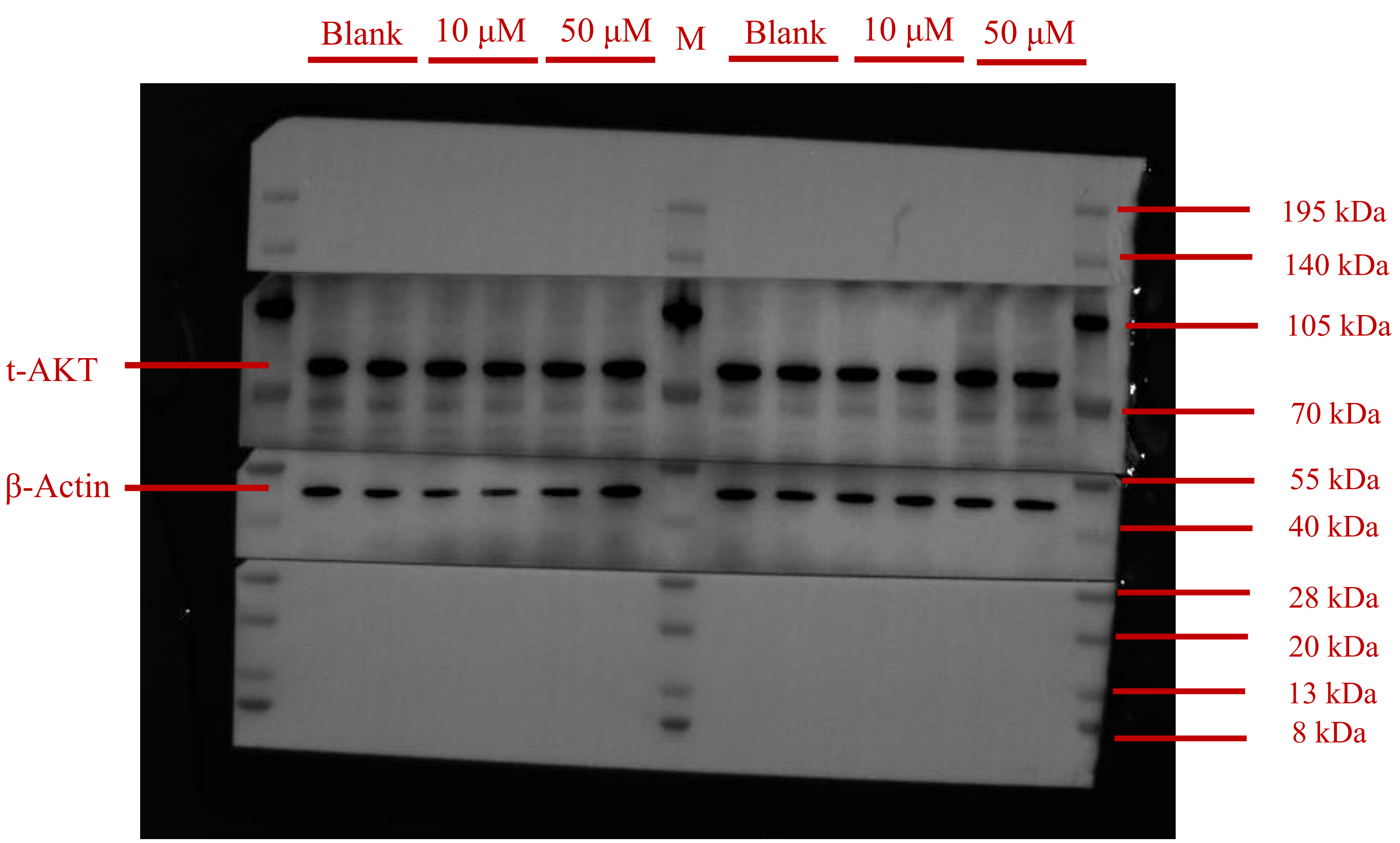

Figure S8 - unmarked

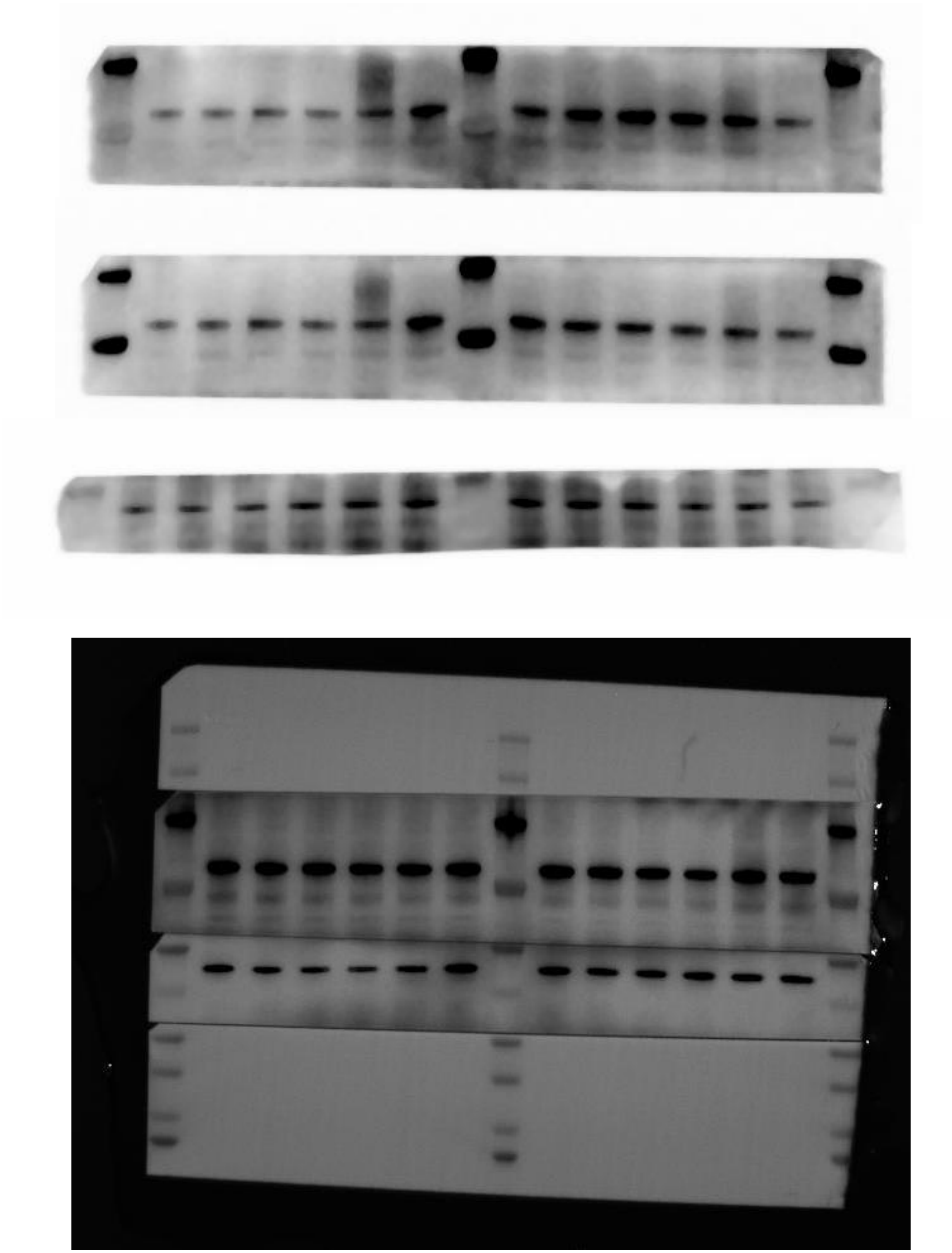

Supplement: Supplementary file 1 [file animals-15-02230-s001.zip › Figures S5-S8.pdf]
