# Supplementary material for: Characterization of the Avian Mitochondrial-Derived Peptide MOTS-c and Its Potential Role as a Metabolic Regulator
Source: Animals (Basel). 2025 Jul 29;15(15):2230. doi: 10.3390/ani15152230 (PMC12345487; doi:10.3390/ani15152230)
Supplement: Supplementary file 1 [file animals-15-02230-s001.zip › Figures S1-S4.pdf]

Supplementary materials

Figure S1. The HPLC test result of chicken MOTS-c.

Figure S2. The MS test result of chicken MOTS-c.

Figure S3. Differentially expressed genes identified using RNA-seq analysis in MOTS-c-treated chicken embryo primary hepatocytes.

Figure S4. Effect of chicken MOTS-c on cell proliferation in primary embryo hepatocytes.

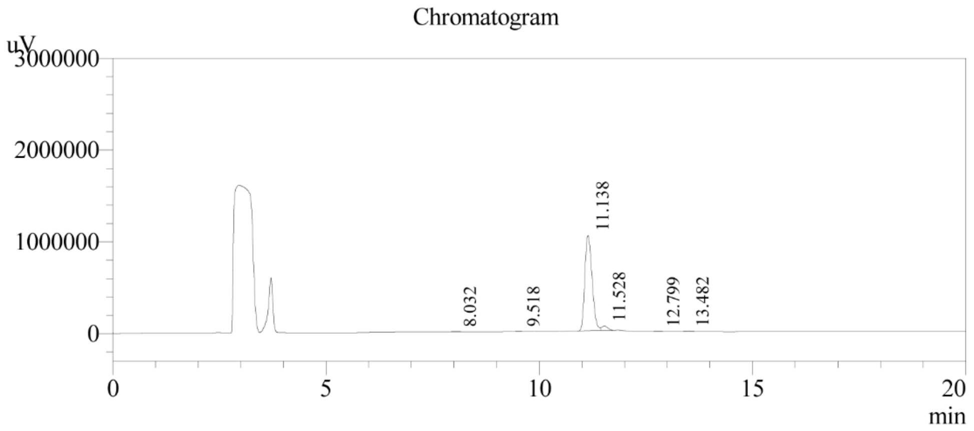

Figure S1. The HPLC test result of chicken MOTS-c.

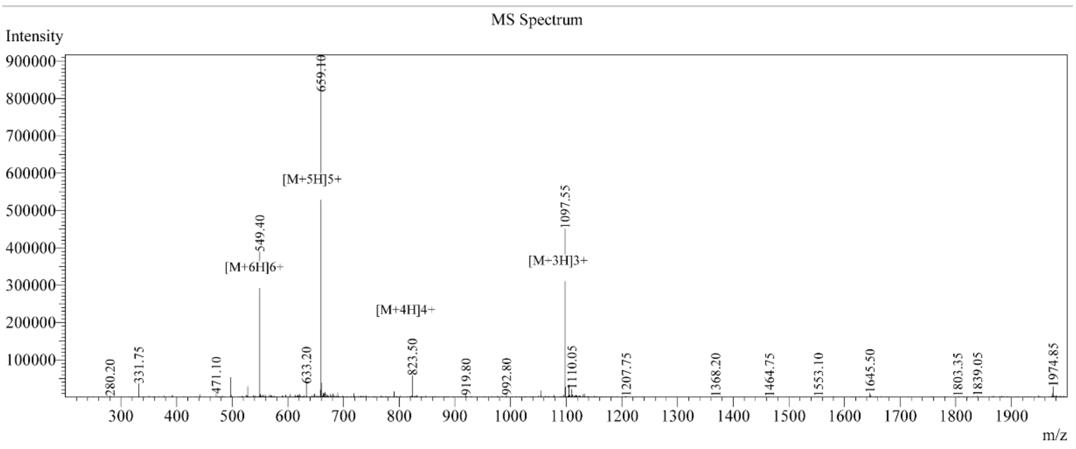

Figure S2. The MS test result of chicken MOTS-c.

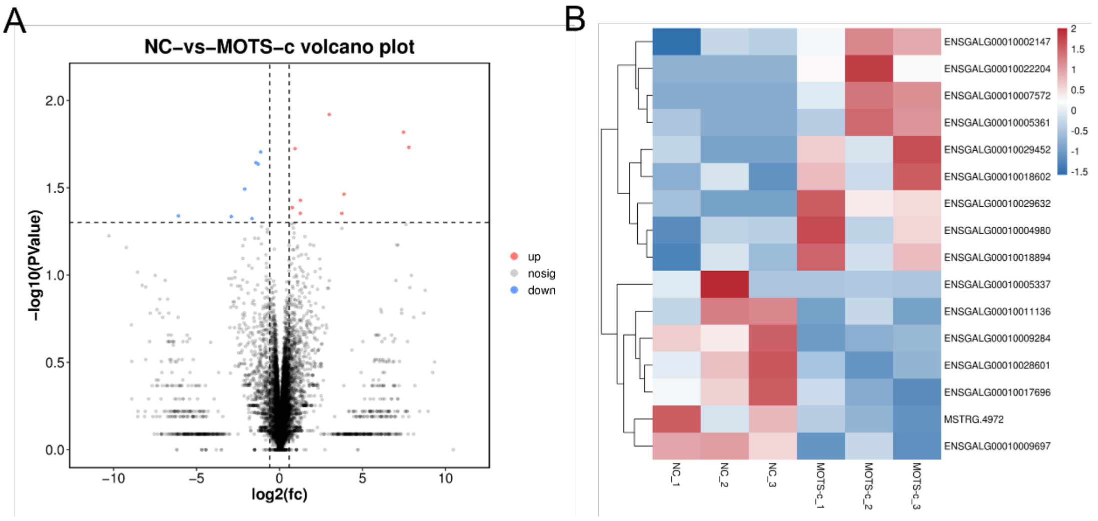

Figure S3. Differentially expressed genes identified using RNA-seq analysis in MOTS-c-treated chicken embryo primary hepatocytes. (A)Volcano plot of

differentially expressed genes (DEGs); (B) Heat-map of DEGs.

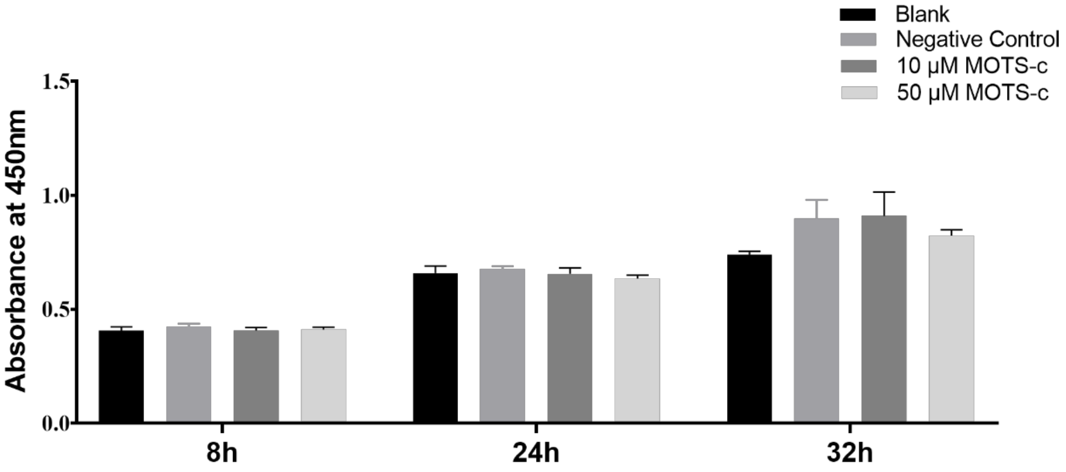

Figure S4. Effect of chicken MOTS-c on cell proliferation in primary embryo hepatocytes. N=8.
